# Use of ifosfamide, carboplatin and etoposide in combination with brentuximab vedotin or romidepsin based on CD30 positivity in relapsed/refractory peripheral T‐cell lymphoma

**DOI:** 10.1002/cnr2.1581

**Published:** 2022-03-08

**Authors:** Cesar Gentille, Humaira Sarfraz, Jitesh Joshi, Jasleen Randhawa, Shilpan Shah, Sai Ravi Pingali

**Affiliations:** ^1^ Houston Methodist Cancer Center Houston Texas USA; ^2^ Houston Methodist Hospital Houston Texas USA

**Keywords:** brentuximab vedotin, ICE, relapsed/refractory PTCL, romidepsin, salvage chemotherapy

## Abstract

**Background:**

Relapsed/refractory peripheral T‐cell lymphoma (R/R PTCL) has a poor prognosis. Romidepsin (Ro) and brentuximab vedotin (Bv), combined with ifosfamide, carboplatin, and etoposide (ICE) has not been significantly studied in PTCL.

**Aim:**

We report outcomes of Bv‐ICE in CD30 (+) and Ro‐ICE in CD30 (−) R/R PTCL treated in “Blinded for peer review” Cancer Center.

**Methods and Results:**

We retrospectively identified R/R PTCL patients treated with BV‐ICE or romidepsin‐ICE from May 2016 to September 2019. Out of 13 R/R PTCL patients, 6 were treated with Bv‐ICE and 7 with Ro‐ICE. Bv‐ICE had an overall response rate (ORR) of 66.7%, with all the patients achieving a complete response. ORR was 71.4% for Ro‐ICE with 57.1% of patients achieving a complete response. Two patients treated with Bv‐ICE and three treated with Ro‐ICE received transplantation.

**Conclusion:**

In our experience, treatment with Bv‐ICE and Ro‐ICE based on CD30 positivity is feasible and effective to treat patients with R/R PTCL.

## 1 INTRODUCTION

Relapsed/refractory peripheral T‐cell lymphoma (PTCL) is a disease with poor prognosis.^1^ There is no standard approach for the treatment of refractory disease. The combination of ifosfamide, carboplatin and etoposide (ICE) has been effective in relapsed/refractory non‐Hodgkin lymphoma (HL) with overall response rates (ORR) of at least 50% in T‐cell lymphomas.^2^ Single‐agent chemotherapy with romidepsin and a CD30‐antibody drug conjugate, brentuximab vedotin (BV), have shown lower response rates in R/R PTCL ranging from 25% to 41%.^3^ Outcomes of the combination of a salvage chemotherapy with either romidepsin or brentuximab vedotin have been scarcely reported. We describe “Blinded for peer review” Cancer Center's experience using Bv‐ICE in CD30 (+) PTCL and romidepsin‐ICE in CD30 (−) PTCL.

## 2 METHODS

We retrospectively identified R/R PTCL patients treated with BV‐ICE or romidepsin‐ICE from May 2016 to September 2019. Our primary objective was to assess the efficacy in terms of ORRs of both salvage chemotherapy regimens. Secondary objectives included duration of response (DOR), hematopoietic cell transplant rate and toxicities.

We queried the medical record database for age, sex, therapy response, date of death/last follow‐up, transplant date as well as laboratory studies including hemoglobin, platelet count, white blood cell count, creatinine, bilirubin, and liver enzymes. We used frequencies and medians to analyze the quantitative variables. Response was assessed by radiologic evidence (CT or PET scan) showing a decrease of tumor burden or resolution of the malignant lesions. Toxicities such as cytopenias, liver and renal function abnormalities were defined per the Common Terminology Criteria for Adverse events version 4.0. The study protocol was reviewed and approved by The Houston Methodist Hospital Institutional Review board.

Chemotherapy with Bv‐ICE and romidepsin‐ICE were given depending on CD30 status. ICE regimen was administered intravenously (IV) using the following doses: Ifosfamide at 5000 mg/m^2^ (continuous infusion over 24 h on day 2), carboplatin with an area under the curve of 5 (day 2) and etoposide at 100 mg/m^2^ (days 1–3). Mesna was given concurrently on day 2. Brentuximab vedotin IV was given at 1.5 mg/kg (days 1 and 8) if CD30 positive and romidepsin IV at 8 mg/m^2^ (days 1 and 4) if CD30 negative. Notably, dose modifications were done per physician's choice. BV‐ICE and romidepsin‐ICE were administered as 21‐day cycles.

## 3 RESULTS

Thirteen patients with R/R PTCL were identified: Six with relapsed and seven with refractory disease. Bv‐ICE was given to six patients and romidepsin‐ICE to seven. The male to female ratio was 5.5: 1. The median age was 65 years old (range: 22–83). More than 75% had stage IV PTCL. The most common subtype was PTCL, not otherwise specified (46.2%) followed by angioimmunoblastic T‐cell lymphoma (30.8%). Out of the six relapsed PTCL patients, median time from initial chemotherapy to relapse was 10.5 months (3–24 months). Further details in Table 1.

**TABLE 1:** Patient characteristics

	Value, *n* = 13
Age (median)	65 (22–83)
Sex	
Male	11
PTCL, classification	
PTCL, NOS	6 (46.2%)
AITL	4 (30.8%)
ALCL	2 (15.4%)
ALCL ALK +	1 (7.7%)
ALCL ALK −	1 (7.7%)
Hepatosplenic	1 (7.7%)
Stage	
III	3 (23.1%)
IV	10 (76.9%)
Performance status (median)	
ECOG	1 (1–2)
IPI at initial diagnosis	
2	5 (38.4%)
3	4 (30.8%)
Unavailable	4 (30.8%)
# of previous regimens (median)	1 (1–2)
CHOP	7 (53.8%)
EPOCH	4 (30.8%)
CHOEP	1 (7.7%)
CHP + Brentuximab vedotin	1 (7.7%)
# of patients with previous stem cell transplant	1 (7.7%)

Abbreviations: AITL, angioimmunoblastic T‐cell lymphoma; ALCL, anaplastic large cell lymphoma; CHOP, cyclophosphamide, doxorubicin, vincristine, and prednisone; CHOEP, cyclophosphamide, doxorubicin, vincristine, etoposide, and prednisone; CHP, cyclophosphamide, doxorubicin, and prednisone; EPOCH, etoposide, prednisone, vincristine, cyclophosphamide, and doxorubicin; PTCL, peripheral T‐cell lymphoma.

Dose reductions were made for four patients receiving Bv (median dose 1.4 mg/kg) and two receiving romidepsin (median dose 8 mg/m^2^). ICE was reduced in six patients for ifosfamide (median dose 3750 mg/m^2^), two for carboplatin (median AUC 5), and six for etoposide (median dose 75 mg/m^2^). All patients got at least 1 dose of G‐CSF (pegfilgastrim, filgastrim, or biosimilar) except for 1 patient in each group; both were not treated with ifosfamide and received less than 2 cycles of chemotherapy.

As detailed in Table 2, Bv‐ICE had an ORR of 66.7% with all patients achieving complete response (CR). ORR was 71.4% for romidepsin‐ICE and CR was 57.1%. The median DOR was 7.5 months for Bv‐ICE and 6 months for romidepsin‐ICE. The median number of cycles given for romidepsin‐ICE was 2 and 3 for Bv‐ICE. Five patients were consolidated with stem cell transplant: 3 in the romidepsin‐ICE group and 2 in the Bv‐ICE group.

**TABLE 2:** Chemotherapy regimen outcomes and toxicities

	Bv‐ICE (*n* = 6)	Ro‐ICE (*n* = 7)
Overall response rate	66.7%	71.4%
Complete remission	66.7%	57.1%
Partial remission	0	14.3%
Progressive disease	33.3%	28.6%
Patients consolidated with stem cell transplant	2 (33.3%)	3 (42.9%)
Autologous	1	2
Allogeneic	1	1
Toxicities ≥ Gr 3		
Anemia	66.7%	100.0%
Thrombocytopenia	50.0%	71.4%
Severe neutropenia (ANC < 500)	83.3%	42.9%
Abnormal liver function tests	33.3%	14.3%
Elevated creatinine	0.0%	0.0%

Abbreviation: ICE, ifosfamide, carboplatin, and etoposide.

Cytopenias (of any grade) occurred in all patients receiving Bv‐ICE and romidepsin‐ICE. Grade 3 and 4 cytopenias were seen more frequently after treatment with romidepsin‐ICE (85.7%) than with Bv‐ICE (66.7%); anemia was the most common cytopenia for both. However, severe neutropenia was seen at a higher rate in patients treated with Bv‐ICE (83.3%) than romidepsin‐ICE (42.9%). Three patients treated with Bv‐ICE had neutropenic fever requiring hospital admission; no patients treated with romidepsin‐ICE were affected.

Overall, metabolic abnormalities were seen at a lower rate than cytopenias. An elevated creatinine and abnormal LFTs (elevated AST/ALT and bilirubin) were seen in two patients (33.3%) treated with Bv‐ICE and two patients (28.6%) treated with romidepsin‐ICE. Three of these events were grade 3 or higher (2 in Bv‐ICE and 1 in romidepsin‐ICE). Peripheral neuropathy was seen in a third of patients receiving Bv‐ICE.

## 4 DISCUSSION

Combination with salvage chemotherapies in R/R PTCL has not been well studied. Our approach was to use the tumor's biology to determine the potential best treatment. Therefore, we would choose between two FDA‐approved drugs for R/R PTCL, romidepsin, and brentuximab vedotin, based on CD30 positivity and combine them with salvage chemotherapy (ICE). Romidepsin, a histone deacetylase inhibitor, targets the epigenome via histone modification and has cytotoxic properties; its ORR is below 40% in phase II studies of R/R PTCL.^3^ Combinations with ICE have been reported previously. In a prospective trial of 18 patients with R/R PTCL, romidepsin‐ICE had an ORR and CR rate of 93% and 80% respectively.^4^ Our response rates were lower; however, transplant rates were similar (close to 50%). Differences in response are likely related to the size of our retrospective sample. Hematological toxicities were comparable with 83% of patients having at least grade 3–4 thrombocytopenia. In addition, less than 20% of patients presented with a liver function abnormality. Overall, our outcomes were better in comparison to romidepsin monotherapy and ICE alone.

Other combinations of romidepsin with salvage chemotherapy have been less successful. In a phase I study, R/R PTCL patients were treated with GDP and romidepsin. From 10 evaluable patients, an ORR of 60% with no CRs was noted. Toxicity higher than grade 2 was significant for myelosuppression and infections (55%–75%).^5^ Combinations with bortezomib, bendamustine, liposomal doxorubicin, and gemcitabine have been disappointing with equivalent or lower ORRs than single agent romidepsin.^6^, ^7^, ^8^, ^9^ Data from phase I studies combining pralatrexate or azacytidine to romidepsin are promising with ORRs (71%–73%) and CRs (50%–55%) comparable to our results.^10^, ^11^

Brentuximab vedotin is an antibody drug conjugate consisting of an anti‐CD30 monoclonal antibody and a microtubule inhibitor, monomethyl auristatin E.^3^ Our study is one of the first ones to report outcomes of Bv‐ICE in R/R PTCL. A prior retrospective study from Van de Wyngaert et al. treated 14 patients with CD30 + R/R PTCL using this regimen however the reported ORR was lower at 29% with a CR rate of 14%.^12^ We believe that patient related factors such as a worse performance status limiting the number of cycles received (median number of cycles was 1) as well as a higher number of prior therapies suggesting a more aggressive disease are the most likely explanations for this finding which was discrepant to our results. In addition, the authors cite CD30 positivity percentage as a potential predictive factor with lower rates showing a suboptimal response to treatment; this variable was not measured in our study, but it may have played a role in the difference between both cohort's ORR. The most common toxicities were infections (37%) and cytopenias (7%–17%); 94% of the cytopenias were grade 3–4 which is higher than our results and may reflect the effect of prior therapies. Only one patient had peripheral neuropathy probably given the low number of cycles given. Additional reports are needed to continue to shed light on the use of BV in association with chemotherapy as salvage therapy in PTCL.

Bv‐ICE has also been effective as a salvage regimen in HL. Two phase I/II studies in relapsed/refractory HL patients showed response rates over 90% with CRs ranging from 69% to 74%; both cohorts had over 40 patients.^13^, ^14^ Transplant rates ranged from 80% to 86% after salvage treatment. Toxicities overall were tolerable and similar to our findings; grade 3–4 hematological toxicity was reported at 71% and infections and febrile neutropenia/sepsis ranged from 11% to 21%. Neuropathy was reported in 36% of patients in one study comparable to our rate; interestingly, the other group did not report any despite having similar populations and receiving at least two cycles of Bv‐ICE.

There have been few studies exploring the combination of brentuximab with other agents for PTCL. A prospective analysis of lenalidomide in combination with BV in 17 patients with relapsed and/or refractory T‐cell lymphoma (three with PTCL, the rest with cutaneous T‐cell lymphoma) showed an ORR of 33% with two CRs and three partial remissions (PR), similar in efficacy to single‐agent BV.^15^ Bendamustine with brentuximab vedotin was studied in a phase 1/2 cohort of refractory HL that included one patient with anaplastic large cell lymphoma who went into PR.^16^ Two retrospective studies showed improved outcomes with this regimen (ORR 60%–78%) in nine patients with cutaneous T‐cell lymphoma and five with PTCL^17^, ^18^; although comparable in ORR, their CR rate out of the total cohort was lower at 35.7% while all of our patients achieved CR. In addition, less than 15% of patients received transplant in comparison to 33.3% in our study.

Even though grade 3–4 anemia and thrombocytopenia were lower with Bv‐ICE in comparison to romidepsin‐ICE, severe neutropenia and neutropenic fever were more common. Studies using romidepsin and brentuximab as single agents reported less than 20% grade 3–4 cytopenias, except for brentuximab which had a neutropenia rate of 21%.^19^, ^20^, ^21^ All grade 3–4 metabolic abnormalities were related to liver function in both of our cohorts. Neither liver nor renal toxicity has been frequently reported in single agent clinical trials (<10%). In studies using combination with multiagent chemotherapy, abnormal liver function was reported with Ro‐ICE at a similar level (11%).^4^ Bv‐ICE had a higher rate of transaminitis/elevated bilirubin (33.3%) in comparison to a prospective study using this combination with no mention of liver toxicity.^13^ This difference could be related to the treated patient population (more likely to have severe disease and comorbidities) as well as the study design (small retrospective cohort with no inclusion criteria). Despite the reported AEs, most of our patients were able to complete more than two cycles of chemotherapy.

In conclusion, treatment with Bv‐ICE and romidepsin‐ICE can be effective salvage regimens for R/R PTCL. Both could improve remission rates prior to transplant with ORRs and CRs over 50%. In our study, more than a third of patients were able to receive consolidation transplantation. Even though the rate of grade 3 and 4 cytopenias were higher than 50% with the combination of romidepsin or brentuximab vedotin with ICE, most patients tolerated treatment well. In our experience, a treatment strategy based on CD30 positivity is feasible and effective to treat patients with R/R PTCL. Further prospective studies are needed to evaluate the promising role of these regimens in this rare disease.

## CONFLICT OF INTEREST

The authors have stated explicitly that there are no conflicts of interest in connection with this article.

## AUTHOR CONTRIBUTIONS

**Cesar Gentille:** Conceptualization (lead); data curation (equal); formal analysis (lead); investigation (lead); methodology (lead); writing – original draft (lead); writing – review and editing (lead). **Humaira Sarfraz:** Data curation (supporting); investigation (supporting); supervision (supporting); writing – review and editing (supporting). **Jitesh Joshi:** Supervision (equal); visualization (equal). **Jasleen Randhawa:** Supervision (equal); visualization (equal). **Shilpan Shah:** Supervision (equal); validation (equal). **Sai Ravi Pingali:** Conceptualization (lead); supervision (lead); validation (lead); writing – review and editing (lead).

## ETHICS STATEMENT

The study protocol was reviewed and approved by The Houston Methodist Hospital Institutional Review board. Need for informed consent in this retrospective study was waived.

## Data Availability

The data that support the findings of this study are available from the corresponding author upon reasonable request.
